# Illinois State Medical Society

**Published:** 1856-07

**Authors:** 


### EDITORIAL.

*Illinois State Medical Society.*

We devote all the space usually assigned to selected matter in the present number of the *Journal*, to the record of proceedings of the State Medical Society, at its recent annual meeting. The meeting in Vandalia was a very pleasant one. The Committee of Arrangements provided a very good room for the accommodation of the Society, and discharged all their duties with fidelity. The whole number in attendance was about *thirty*, mostly from the middle and southern counties; indeed, we do not recollect the presence of a single delegate from the northern part of the State, except the *four* from Chicago. We think those members of the Society at Joliet, Ottawa, Rockford, Mt. Morris, &c., who had taken an active part in the previous meetings, committed a great mistake in not going to Vandalia. They thereby lost the opportunity of visiting one of the most pleasant parts of the State, and of forming the acquaintance of a larger number of physicians from the southern counties than at any previous meeting.

The proceedings of the meeting were characterized by the most cordial good feeling, and close attention to the proper business of the Society. The transactions will be published at an early day, and will contain matter of interest to every physician in the State.

